# Serum Iodine as a Potential Individual Iodine Status Biomarker: A Cohort Study of Mild Iodine Deficient Pregnant Women in China

**DOI:** 10.3390/nu15163555

**Published:** 2023-08-11

**Authors:** Xueqing Li, Pengcheng Tu, Simeng Gu, Zhe Mo, Lizhi Wu, Mingluan Xing, Zhijian Chen, Xiaofeng Wang

**Affiliations:** Department of Environmental Health, Zhejiang Provincial Center for Disease Control and Prevention, 3399 Binsheng Road, Hangzhou 310051, China; xqli@cdc.zj.cn (X.L.); pchtu@cdc.zj.cn (P.T.); smgu@cdc.zj.cn (S.G.); zhmo@cdc.zj.cn (Z.M.); lzhwu@cdc.zj.cn (L.W.); mlxing@cdc.zj.cn (M.X.); zhjchen@cdc.zj.cn (Z.C.)

**Keywords:** serum iodine, urinary iodine, pregnant women, individual iodine status, thyroid diseases, thyroid function

## Abstract

Iodine deficiency during pregnancy is a widespread public health concern, but indicators and methods for assessing iodine nutritional status are lacking. Serum iodine concentration (SIC) is an important iodine metabolism biomarker and can, to some extent, predict the risk of thyroid diseases, making it a potential biomarker for assessing individual iodine nutrition levels. Our study aimed to analyze the relationship between SIC and thyroid function in a cohort of mild iodine deficient pregnant women in China in order to explore the potential of SIC as a biomarker of individual iodine status in pregnancy. A total of 1540 early pregnant women (gestation < 10 weeks) aged 18 to 45 years old were included in the final study from a Zhejiang multicenter population-based mother and child cohort. Repeated measures of SIC, urinary iodine concentration (UIC), and thyroid function were taken at approximately 10, 17, and 32 weeks of gestation. The SIC was statistically correlated with all thyroid function indexes in the first trimester, and a very strong positive correlation with FT4 over three trimesters (r = 0.449, 0.550, and 0.544, respectively). Pregnant women with an SIC < 72.4 µg/L were at a higher risk of hypothyroxinemia (adjusted OR = 8.911, 95% CI = 5.141–15.447) and iodine deficiency (adjusted OR = 1.244, 95% CI = 1.031–1.502), while those with an SIC > 93.9 µg/L were at a higher risk of thyrotoxicosis (adjusted OR = 11.064, 95% CI = 6.324–19.357) and excessive iodine (adjusted OR = 11.064, 95% CI = 6.324–19.357). In contrast, the UIC was not correlated with thyroid diseases (*p* > 0.05). These findings indicate that the SIC is a potential biomarker for assessing individual iodine nutrition and thyroid dysfunction in pregnant women.

## 1. Introduction

Iodine is an essential micronutrient for the synthesis of the thyroid hormone, with a key role in the regulation of body growth and basic metabolism. Inadequate iodine intake can lead to iodine deficiency disorder (IDD), which affects all stages of the human life cycle, especially during pregnancy. Moreover, pregnant women require about 50% more iodine than non-pregnant women due to increased iodine loss resulting from increased thyroid hormone secretion and maternal glomerular filtration, and the fetal iodine requirement for thyroid hormone production [1,2]. The higher iodine requirement renders pregnant women at higher risk of iodine deficiency. Iodine deficiency during pregnancy leads to infertility, gestational hypertension, abortions, premature birth, stillbirth, congenital malformations, and can even stunt the brain and nerve development of the fetus [3]. Therefore, maintaining an adequate intake of iodine is essential for pregnant women.

Before the 1970s, China had a severely iodine-deficient population. In 1995, the universal salt iodization (USI) program was introduced nationwide, and legislation was enacted requiring iodine to be added to table salt. Iodine nutrition has been greatly improved nationwide, but iodine deficiency still exists among vulnerable populations, especially pregnant women [4,5,6]. According to the 2014 IDD surveillance data, two-thirds of Chinese provinces have insufficient iodine levels among pregnant women [7]. The criteria for assessment of the IDD are based on the median urinary iodine concentration (UIC) recommended by the World Health Organization (WHO) [8]. Although UIC is the most commonly used biomarker for assessing iodine status in populations [9], UIC only reflects recent iodine intake. It can be affected by water intake and diet, especially during pregnancy, as excessive water intake leads to an increased urine output and dilution of iodine concentrations. Therefore, UIC may not be the most accurate indicator of the iodine nutritional status, especially for pregnant women, and a sensitive biomarker for assessing the individualized iodine nutrition of pregnant women is urgently needed.

In addition to UIC, thyroid volume, thyroid function, and serum iodine concentration (SIC) have also been used as sensitive biomarkers to assess the iodine nutrition status. Regarding thyroid volume indicators, ultrasounds can accurately measure thyroid volume, but ultrasounds require specialized operators, and a goiter usually occurs after several months of iodine deficiency. Therefore, the thyroid volume can only reflect longer-term iodine nutrition status [10]. Although thyroid function has functional indicators, they are not sensitive markers of individual iodine status; owing to tight homeostatic regulation, their values can be maintained within the normal reference range in the presence of inadequate iodine intake [11]. By contrast, the SIC is a key biomarker of iodine metabolism, as serum iodine is controlled by intrinsic mechanisms. However, long-term inadequate iodine status will break the iodine balance, leading to abnormal serum iodine levels and ultimately thyroid dysfunctions and diseases. Thus, the SIC may be a potential candidate as a biomarker for the evaluation of individual iodine status [12,13,14,15].

Currently, there is no accurate method to reflect individual iodine nutrition levels in pregnant women. Most studies have focused on the application of the SIC as an iodine metabolism biomarker in patients with thyroid diseases, but its application in population studies, especially in pregnant women, remains elusive. The relationship of the SIC and iodine status or thyroid diseases has been preliminarily explored within limited populations (adults and school-age children) [15,16], but there has been limited exploration of this relationship among the pregnant population [17]. Particularly in relation to cohort study designs, there are no longitudinal studies on SIC data from pregnant women in China. Therefore, in the present study, we aimed to analyze the relationship of the SIC, UIC, and thyroid function in a cohort of pregnant women in China in order to explore the potential of SIC as a sensitive biomarker reflecting individual iodine status in pregnant women with mild iodine deficiency.

## 2. Materials and Methods

### 2.1. Study Population

This study was a prospective observational study from a Zhejiang multicenter population-based mother and child cohort. Between August 2019 and December 2021 we recruited participants from five representative cities in Zhejiang: Hangzhou (north), Quzhou (west), Jinhua (central), Zhoushan (east), and Taizhou (south). All participants were recruited at their first routine antenatal care visit in the local maternal and child healthcare hospital. The inclusion criteria were that the participant be (a) aged at 18–45 years; (b) gestation < 10 weeks; (c) residing locally for more than 1 year and planning to stay there for the following 3 years. Relevant exclusion criteria were taking iodine supplementation, being on iodine-containing contrast media within one year, and with a history of thyroid disease or other chronic diseases. Altogether, 1832 participants met these criteria and were included in this cohort study. Each participant was required to complete a questionnaire at their first routine antenatal care visit (gestation <10 weeks), and then blood and urine samples were collected at approximately 10, 17, and 32 weeks of gestation. Finally, 1540 pregnant women were included in the final study after excluding participants missing blood or urine samples at the first trimester (*n* = 292).

The study was conducted in accordance with the Declaration of Helsinki and approved by the Ethics Committee of Zhejiang Provincial Center for Disease Control and Prevention. Written informed consent was signed by all participants.

### 2.2. Laboratory Analysis

#### 2.2.1. Sample Collection and Processing

Fasting blood and spot-urine samples were collected from each participant at three follow-up visits, at approximately 10, 17, and 32 weeks of gestation, and thus we obtained measurements from the first, second, and third trimester. Serum was separated via centrifugation for SIC and thyroid function detection. To avoid iodine contamination during blood collection, alcohol disinfection was used instead of iodophor disinfection. Urine samples were collected in sterile urine cups for UIC detection. All samples were sub-packed and stored frozen at −80 °C and analyzed within one month of collection.

#### 2.2.2. Testing and Analysis

UIC was measured using the China Health Standard Method for Determination of Iodine in Urine based on the As3+–Ce4+ Catalytic Spectrophotometry (WS/T 107.1-2016). The Chinese National Reference Laboratory for Iodine Deficiency Disorders provided the external quality control samples. SIC was measured using inductively coupled plasma mass spectrometry (ICP-MS; 7900, Agilent Technologies Ltd, Tokyo, Japan. An amount of 100 µL serum aliquot was added to a 1.9 mL diluent containing Triton X-100 (0.1%) and nitric acid (1.0%). A number of quality control checks were performed for each 20-sample batch. The quality control checks included blank, duplicate, standard reference material, and quality control sample (Standard lyophilized human serum reference material, Trace Elements Serum L-2, Seronorm, Norway). 90–110% of iodine was recovered in standard reference material and the quality control results were within confidence intervals. Serum thyroid-stimulating hormone (TSH), free triiodothyronine (FT3), free thyroxine (FT4), thyroglobulin (Tg), thyroglobulin antibody (TgAb), and thyroid peroxidase antibody (TPOAb) were measured using an electrochemiluminescence immunoassay with a Cobas e411 analyzer (Roche Diagnostics GmbH, Mannheim, Germany), coupled with corresponding calibration materials, reagents, and quality controls. The quality control procedure was carried out in accordance with the manufacturer’s instructions before, during, and after the testing. To ensure the reliability of the results, all samples were analyzed after testing of the quality control samples.

Thyroid diseases during pregnancy, including clinical hypothyroidism, subclinical hypothyroidism, hypothyroxinemia, and thyrotoxicosis, were adopted from the Guidelines on Diagnosis and Management of Thyroid Disease during Pregnancy and the Postpartum (2nd edition, Chinese Society of Endocrinology and Chinese Society of Perinatal Medicine, 2019). The laboratory reference ranges obtained using a Cobas e411 (Roche Diagnostics GmbH, Mannheim, Germany) system are summarized in Table 1. A positive TPOAb was defined as that with a value ≥ 34 IU/mL and a positive TgAb as that with a value ≥ 115 IU/mL.

### 2.3. Assessment of Covariates

Each participant was required to complete a questionnaire to collect information, including age, gestational week, education, alcohol use during pregnancy, and active and passive smoking during pregnancy. Gestational age was calculated using the last menstrual period. The weight and height were measured at their first routine antenatal care visit (gestation < 10 weeks). Body mass index (BMI) was estimated by dividing weight (kg) by height squared (m^2^).

### 2.4. Statistical Analysis

All statistical analyses and data processing were conducted using SPSS 19.0 (IBM, Inc., New York, NY, USA), Microsoft Excel (Win11 2021), and GraphPad Prism 8.0 software from GraphPad Software, Inc. The Kolmogorov–Smirnov method was used to test continuous variable distribution normality. Normally distributed data are expressed as the mean ± standard deviation (SD), and non-normally distributed data are expressed as the median with the interquartile range (IQR: 25th–75th percentiles). The SIC, UIC, and thyroid function were assessed using the Kruskal–Wallis test in groups, and pairwise comparisons were performed using the Mann–Whitney rank sum test. Categorical variables were expressed as counts and percentages, and comparisons between the groups were made using the chi-square (χ2) test or Fisher’s exact test. The Spearman correlation was used to test associations among the SIC, UIC, and thyroid function, and correlation coefficients (r) were calculated.

Binary logistic regression analysis was used to explore associations between the SIC, UIC, and thyroid diseases. The results were expressed as odds ratios (ORs) and 95% confidence intervals (CIs) for thyroid disease and iodine status. The following variables were selected as covariates to estimate adjusted ORs and 95% CIs: age (continuous), BMI (continuous), education (junior high school or below/high school/college or above), and smoking status (yes/no). Covariates were selected for inclusion in multivariable models based on univariate analysis. All figures are two-tailed cutoffs, and significance was set at *p* < 0.05, unless otherwise stated.

## 3. Results

### 3.1. Participant Characteristics

The baseline characteristics of 1540 pregnant women were collected at their first routine antenatal care visit (gestation < 10 weeks), as shown in Table 2. The mean age and BMI were 29.0 ± 4.6 years and 21.7 ± 3.3 kg/m^2^, respectively. About 86% of the pregnant women were aged 23–35 years. Among the pregnant women, 54.9% had a college degree or above, 4.5% used alcohol during pregnancy, 1.2% smoked during pregnancy, and 20.3% were passively exposed to second-hand smoke during pregnancy.

### 3.2. Distribution of Thyroid Function, Urinary Iodine, and Serum Iodine in Pregnant Women by Trimester

Table 3 shows the thyroid function, UIC, and SIC of pregnant women, stratified by trimester. A significant increasing trend was found in the TSH across three trimesters (*p* < 0.001), whereas serum FT3 and FT4 values decreased (*p* < 0.001). A pairwise comparison of the Mann–Whitney rank sum test showed that Tg was significantly higher in the third trimester than in the second trimester (*p* = 0.006), but no significant differences were present among the other trimesters. The median SIC was 79.6, 86.5, and 83.7 µg/L in the first, second, and third trimesters, respectively. SIC had significant differences between pregnant women at varying trimesters (*p* < 0.001), while the median SIC was the highest in the second trimester. Likewise, SIC was significantly higher in the third trimester than in the first trimester (*p* < 0.001).

UIC was significantly higher in the first and second trimesters than in the third trimester (*p* = 0.003 and 0.005, respectively), but no significant difference between the first and second trimesters was present (*p* = 0.813). The median UIC values for pregnant women in the first and second trimesters were 112.9 µg/L and 114.0 µg/L, respectively. This was decreased to 105.0 µg/L in the third trimester. Therefore, pregnant women in all trimesters were classified as mildly deficient according to the WHO criteria.

We observed significant differences in the prevalence of hypothyroxinemia and thyrotoxicosis among pregnant women of different trimesters (*p* < 0.001), with the highest prevalence being among women in the first trimester. In addition, the positive rates of TgAb and TPOAb in the first and second trimesters were significantly higher than that in the third trimester (*p* = 0.001 and *p* < 0.001, respectively). Other abnormal thyroid functions showed no significant differences among the three trimesters.

### 3.3. Correlation between Iodine Levels and Thyroid Function

To investigate the correlation among the SIC, UIC, and thyroid function indexes, Spearman correlation coefficients (r) were calculated, as shown in Table 4. The SIC was statistically significant correlated with all thyroid function indexes in the first trimester. In addition, the SIC showed very strong positive correlations with FT4 in three trimesters (r = 0.449, 0.550, and 0.544, respectively). In contrast, the UIC was not significantly correlated with thyroid function indexes in the first and second trimesters, and the UIC only showed very weak correlations with TSH and Tg in the third trimester.

**Figure 1:**
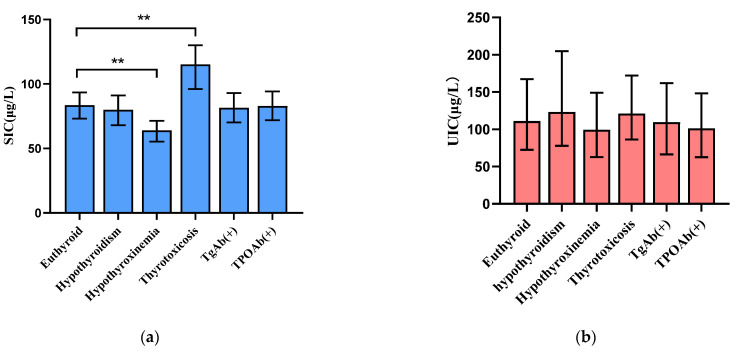
Distribution of iodine levels according to different thyroid disfunctions. Blue: (**a**) SIC, Red: (**b**) UIC. Hypothyroidism includes both clinical hyperthyroidism and subclinical hyperthyroidism, due to the small size in these two thyroid disfunctions. ** *p* < 0.001 the compared euthyroid and thyroid abnormalities groups by the Mann–Whitney rank sum test. Abbreviations: TgAb, thyroglobulin antibody; TPOAb, thyroid peroxidase antibody; UIC, urinary iodine concentration; SIC, serum iodine concentration.

Furthermore, we analyzed the median concentrations of the SIC and UIC varied in different thyroid dysfunctions, as shown in Figure 1. Compared with euthyroid pregnant women, hypothyroxinemia pregnant women had a lower median SIC (*p* < 0.001), whereas the median SIC of thyrotoxicosis pregnant women was higher. In addition, there were no significant differences in the median SIC of other thyroid dysfunction pregnant women between the groups of euthyroid pregnant women. However, the median UIC of thyroid dysfunction pregnant women was not significantly different between that of euthyroid pregnant women.

Further binary logistic regression analysis of SIC and UIC was associated with odds of having thyroid diseases (Table 5). When the SIC was treated as quartiles, pregnant women with low SIC levels (quartile 1) were at a higher risk of hypothyroxinemia (unadjusted OR = 9.619, 95% CI = 5.567–16.619). After adjusting for age, BMI, education, and smoking status, the association was changed slightly, but remained significant (adjusted OR = 8.911, 95% CI = 5.141–15.447). Similarly, in unadjusted models, pregnant women with high SIC levels (quartile 4) were at a higher risk of thyrotoxicosis (unadjusted OR = 11.143, 95% CI =6.376–19.473). This verse association was persisted (adjusted OR = 11.064, 95% CI = 6.324–19.357). The UIC was divided into three levels according to the recommended WHO criteria, and associations between the UIC and thyroid diseases were not significant (*p* > 0.05).

Table 6 shows the association between the SIC and iodine status by logistic regression analysis. The adjusted OR for iodine deficiency (UIC < 150 µg/L) in pregnant women with low SIC levels (quartile 1) was 1.244. Pregnant women with high SIC levels (quartile 4) were at a higher risk for excessive iodine (UIC > 500 µg/L) (adjusted OR = 1.866, 95% CI = 1.024–3.399).

## 4. Discussion

The indicators and methods for assessing individual iodine nutritional status are a matter of debate. The most commonly used tool is UIC, which is typically employed to assess the iodine nutritional status of a population. However, UIC is influenced by factors such as water intake, diet, and pregnancy, and these factors can notably skew the results. For instance, pregnant women tend to increase their water intake, leading to significant variations in UIC that do not truly reflect their iodine nutritional status. Despite this, some studies have found that the accuracy of iodine nutrition assessment can be improved by measuring UIC in ten spot-urine samples or 24-hour urine samples. However, these methods pose considerable challenges in terms of sample collection, making them difficult to apply in large-scale epidemiological surveys [18]. Additionally, some studies have attempted to mitigate the effects of urine volume on UIC by adjusting for urinary creatinine concentration [19,20]. Nevertheless, factors such as age, ethnicity, skeletal muscle content, and protein intake can all affect urinary creatinine concentration [21,22]. Therefore, there remains significant controversy over the use of creatinine-adjusted UIC to assess individual iodine nutritional status. Consequently, there is a lack of universally recognized appropriate indicators and methods for evaluating individual iodine nutrition in clinical settings. In particular, for key populations in IDD monitoring, such as pregnant women, iodine deficiency during pregnancy can lead to insufficient thyroid hormone secretion, which affects the thyroid function of both the pregnant woman and the fetus, resulting in impaired fetal neural development. If the window for growth and development is missed, subsequent iodine supplementation cannot undo the adverse effects on the fetus’s intellectual and physical development. Therefore, it is of paramount importance to accurately assess the iodine nutritional status of pregnant women, providing a new scientific basis for tailoring iodine supplementation for individual needs in early life.

SIC is an important biomarker of iodine metabolism. In blood, there is no free iodine, as it is immediately captured by thyroid cells; hence, SIC can directly reflect the bioavailable iodine in the thyroid, representing the actual internal iodine level. It is able to accurately reflect the recent iodine nutritional status of the organism and it does not immediately change with the intake of dietary iodine, making it more stable compared to UIC [14,23]. Long-term inappropriate iodine intake can lead to abnormal SIC levels, thereby affecting thyroid function. Therefore, SIC can, to some extent, predict the risk of thyroid diseases in high-risk populations, making it a potential biomarker for assessing individual iodine nutrition levels [16]. Accordingly, our study focused on the application of SIC in research involving pregnant women, assessing the correlation between SIC, UIC, and thyroid function as a basis for further evaluating the iodine nutritional status of individual pregnant women.

Our study found that in early pregnancy, SIC is correlated with thyroid function indicators (TSH, FT3, FT4, and Tg), and has a strong positive correlation with FT4 throughout all three trimesters. This can be explained by the close correlation between SIC and bioavailable iodine in the thyroid [12,18]. Early pregnancy is a period particularly susceptible to the impact of iodine deficiency. As the thyroid hormones needed by the fetus during this period are provided entirely by the mother, iodine deficiency in the mother at this time can cause irreversible damage to the development of the fetal brain [24]. Therefore, our results also suggest that SIC has good predictive ability for evaluating thyroid function in early pregnancy.

Since both low and high iodine intake may lead to thyroid-related diseases, we also analyzed the relationship between SIC, UIC, and thyroid diseases. The results found that pregnant women with thyrotoxicosis had higher SIC levels than those with normal thyroid function, whereas pregnant women with hypothyroxinemia had lower SIC levels. When the SIC is above the fourth quartile, the risk of thyrotoxicosis in pregnant women is higher, and when the SIC is below the first quartile, the risk of hypothyroxinemia in pregnant women increases. To enhance the reliability of the results, the data were rechecked using Poisson regression with consistent results (Supplementary Table S1). It shows that SIC has good diagnostic value for thyrotoxicosis and hypothyroxinemia. This is consistent with the results of a study on pregnant women in a region in China with appropriate iodine levels [17]. Autoimmune hyperthyroidism is a common cause of thyrotoxicosis in pregnant women, and it has been found that increased iodine intake is linked to a higher prevalence of autoimmune thyroid disease [25]. On the other hand, research has found that iodine deficiency may lead to maternal thyroxinemia [25,26]. Therefore, SIC levels can reflect iodine intake and can be used to evaluate the iodine nutritional status of pregnant women. Our study also supports this conclusion, indicating that pregnant women with low SIC levels are at a higher risk of iodine deficiency (UIC < 150 µg/L), while those with high SIC levels are at a higher risk of excessive iodine (UIC > 500 µg/L). These results highlight the prospects of using SIC as a biomarker for the iodine nutritional status of individual pregnant women.

Our study showed that UIC in the first, second, and third trimester was 112.9, 114.0, and 105.0 µg/L, respectively, all of which were below the suitable level of iodine nutrition recommended by the WHO (150 µg/L), suggesting that pregnant women in the Zhejiang province are mildly iodine deficient. This is consistent with the epidemiological surveillance results in the Zhejiang province from 2015 to 2022. Our study found that UIC had no correlation with thyroid function indicators or thyroid diseases. However, a recent study showed that the relationship between the prevalence of UIC and thyroid diseases embodied a U-shaped curve [27]. This may be due to the fact that there are many influencing factors on UIC in pregnant women, and individual UIC levels fluctuate greatly, so they cannot accurately reflect the actual iodine nutritional status of individual pregnant women. A recent study in Denmark confirmed this hypothesis, finding significant variation in the UIC from urine samples collected from the same group of pregnant women at different locations (hospitals and homes) [28]. Furthermore, UIC levels in pregnant women may also vary by trimester [29]. However, the trend of these changes is controversial: some reports suggest a decrease with gestational age [30], some suggest an increase [31], and others report no change throughout pregnancy [32]. Moreover, the UIC also changes with dietary changes throughout different seasons [33]. These diverse changes in UIC levels can directly affect the evaluation results of iodine nutrition during pregnancy, thus increasing the difficulty of evaluating iodine nutrition during pregnancy. Therefore, our results confirm that UIC is more suitable for assessing population iodine nutrition status, and SIC may be a better biomarker for assessing individual iodine nutrition status in pregnant women.

One of the strengths of this study is that it observed the SIC levels of mildly iodine-deficient pregnant women in China from a longitudinal cohort perspective, analyzing changes in the SIC throughout pregnancy. This effectively controlled for confounding factors due to individual differences such as age and weight among pregnant women, marking it the first such study conducted among pregnant women in the Zhejiang province. This study provides specific SIC reference ranges for different pregnancy periods in pregnant women in the Zhejiang province, with SIC ranges for the first, second, and third trimesters being 54.4–114.3 μg/L, 60.2–112.7 μg/L, and 60.2–110.5 μg/L, respectively. This is lower than the previously reported SIC range for pregnant women in an iodine-sufficient region of China (first trimester: 78.6–178.8 μg/L, second trimester: 67.3–163.8 μg/L, third trimester: 60.2–144.1 μg/L) [17], and is similar to the median SIC range of 67.9–84.8 μg/L, reported in a study of the general population in an iodine-deficient region in China [16].

Our study has some limitations. First, we did not include iodine intake in our study. This information will be evaluated in future research. Second, our study has found that in early pregnancy, the SIC has a good correlation with thyroid function, but due to the limited number of thyroid disease cases, no analysis of the SIC and thyroid disease has been conducted. This requires further exploration with the collection of more early pregnancy samples. Third, our study only collected spot urine, and lacked 24 h urine or adjusted UIC data. However, our study was based on large-scale epidemiological surveys, and these methods pose greater difficulties in terms of sample collection. Lastly, this study only involves pregnant women, the obtained results need to be confirmed in the general population due to differences regarding pregnant women and the general population. Thus, caution should be used when extending our result to other populations. Currently, our group is conducting a large population-based cohort study (*n* = 20,000 participants). Further studies could validate the reliability and applicability of SIC for assessing individual iodine status in the general population. Additionally, it also can provide more extensive data support for establishing reference intervals for serum iodine levels.

## 5. Conclusions

Serum iodine has shown promise as a biomarker for assessing individual iodine nutrition and thyroid dysfunction in pregnant women. This study is the first to report the 95% medical reference range of serum iodine in pregnant women in the Zhejiang province, identifying a correlation between the SIC and thyroid function in pregnant women. A higher SIC was associated with an increased risk of thyrotoxicosis and excessive iodine, while a lower SIC was associated with an increased risk of hypothyroxinemia and iodine deficiency. Overall, SIC has good diagnostic value for thyroid dysfunction.

## Figures and Tables

**Table 1:** Diagnostic criteria for thyroid diseases by trimester ^1^.

	First Trimester	Second Trimester	Third Trimester
Clinical hypothyroidism	TSH > 4.52 mU/L and FT4 < 13.15 pmol/L	TSH > 4.32 mU/L and FT4 < 9.77 pmol/L	TSH > 4.98 mU/L and FT4 < 9.04 pmol/L
Subclinical hypothyroidism	TSH > 4.52 mU/L and 13.15 < FT4 < 20.78 pmol/L	TSH > 4.32 mU/L and 9.77 < FT4 < 18.89 pmol/L	TSH > 4.98 mU/L and 9.04 < FT4 < 15.22 pmol/L
Hypothyroxinemia	0.09 < TSH < 4.52 mU/L and FT4 < 13.15 pmol/L	0.45 < TSH < 4.32 mU/L and FT4 < 9.77 pmol/L	0.30 < TSH < 4.98 mU/L and FT4 < 9.04 pmol/L
Thyrotoxicosis	TSH < 0.09 mU/L and FT4 > 20.78 pmol/L	TSH < 0.45 mU/L and FT4 > 18.89 pmol/L	TSH < 0.30 mU/L and FT4 > 15.22 pmol/L

^1^ Diagnostic criteria based on the Guidelines on Diagnosis and Management of Thyroid Disease during Pregnancy and the Postpartum (2nd edition, Chinese Society of Endocrinology and Chinese Society of Perinatal Medicine, 2019). Abbreviations: TSH, thyroid stimulating hormone; FT4, free thyroxine.

**Table 2:** Baseline (gestation < 10 weeks) characteristics in pregnant women (*n* = 1540).

Characteristic ^1^	Mean ± SD/N(%)
Age, years	29.0 ± 4.6
BMI, Kg/m^2^	21.7 ± 3.3
Education	
Junior high school or below	271 (17.6)
High school	423 (27.5)
College or above	846 (54.9)
Alcohol use during pregnancy	
Yes	69 (4.5)
No	1471 (95.5)
Smoking during pregnancy	
Yes	18 (1.2)
No	1522 (98.8)
Passive smoking during pregnancy	
Yes	313 (20.3)
No	1227 (79.7)

^1^ Continuous data are expressed as the mean ± SD; categorical data are presented as N (%). Abbreviations: SD, standard deviation; BMI, body mass index.

**Table 3:** Thyroid function, urine iodine concentration (UIC), and serum iodine concentration (SIC) in pregnant women by trimester.

	First Trimester (*n* = 1540)	Second Trimester (*n* = 1487)	Third Trimester (*n* = 1306)	*p*
Gestational age (week) ^a^	10 (6, 13)	17 (16, 27)	32 (28, 36)	-
TSH (mIU/L) ^b^	1.1 (0.6, 1.8) **	1.7 (1.2, 2.3) **	1.8 (1.3, 2.4) **	<0.001
FT3 (pmol/L) ^b^	4.8 (4.4, 5.3) **	4.3 (4.0, 4.7) **	3.8 (3.5, 4.2) **	<0.001
FT4 (pmol/L) ^b^	16.7 (15.2, 18.5) **	13.9 (12.7, 15.2) **	12.1 (11.0, 13.3) **	<0.001
Tg (µg/L) ^b^	11.1 (6.6, 17.9)	10.8 (6.2, 17.5) *	11.8 (6.7, 18.3)	0.019
Thyroid dysfunction *n*(%)				
Overt hypothyroidism	7 (0.5)	1 (0.1)	0 (0.0)	-
Subclinical hypothyroidism	22 (1.4)	31 (2.1)	16 (1.2)	0.158
Hypothyroxinemia	51 (3.3)	16 (1.1) ^#^	24 (2.4) ^#^	<0.001
Thyrotoxicosis	76 (4.9)	10 (0.7) ^#^	9 (0.7) ^#^	<0.001
TgAb(+) *n*(%)	114 (7.4) *	82 (5.5) *	21 (1.6)	<0.001
TPOAb(+) *n*(%)	137 (8.9) *	118 (7.9) *	68 (5.2)	0.001
UIC (µg/L) ^b^	112.9 (73.5, 172.2) *	114.0 (73.7, 168.0) *	105.0 (68.2, 156.6)	0.007
SIC (µg/L) ^b^	79.6 (68.1, 91.5) **	86.5 (76.2, 96.5) **	83.7 (73.5, 93.3) **	<0.001

^a^ Values are expressed as the median (min, max). ^b^ Values are expressed as the median (25, 75th percentile). * Compared with the third trimester group, the difference was statistically significant. ^#^ Compared with the first trimester group, the difference was statistically significant. ** The difference was statistically significant between the three trimesters. Abbreviations: TSH, thyroid-stimulating hormone; FT3, free triiodothyronine; FT4, free thyroxine; Tg, thyroglobulin; TgAb, thyroglobulin antibody; TPOAb, thyroid peroxidase antibody; UIC, urinary iodine concentration; SIC, serum iodine concentration.

**Table 4:** Spearman correlation analysis of serum iodine concentration (SIC), urine iodine concentration (UIC), and thyroid function by trimester.

	TSH	FT3	FT4	Tg
First trimester				
UIC	−0.036	−0.049	0.038	−0.018
SIC	−0.282 **	0.178 **	0.449 **	0.108 **
Second trimester				
UIC	0.030	−0.018	0.013	−0.020
SIC	0.024	0.165 **	0.550 **	0.040
Third trimester				
UIC	0.075 **	0.010	0.045	−0.096 **
SIC	−0.041	0.022	0.544 **	0.065 *

** *p* < 0.001, * *p* < 0.05. Abbreviations: TSH, thyroid-stimulating hormone; FT3, free triiodothyronine; FT4, free thyroxine; Tg, thyroglobulin; UIC, urinary iodine concentration; SIC, serum iodine concentration.

**Table 5:** Binary logistic regression exploring the effect of serum iodine level and urinary iodine level on thyroid diseases.

	Hypothyroxinemia				Thyrotoxicosis			
	Unadjusted		Adjusted ^1^		Unadjusted		Adjusted ^1^	
	OR (95% CI)	*p*	OR (95% CI)	*p*	OR (95% CI)	*p*	OR (95% CI)	*p*
SIC (µg/L)								
<72.4 (Quartile 1)	9.619 (5.567–16.619)	0.000	8.911 (5.141–15.447)	0.000	0.400 (0.116–1.386)	0.149	0.415 (0.120–1.440)	0.166
72.4–93.9 (Quartile 2 + Quartile 3)	1 (referent)		1 (referent)		1 (referent)		1 (referent)	
>93.9 (Quartile 4)	0.379 (0.110–1.302)	0.123	0.388 (0.113–1.338)	0.134	11.143 (6.376–19.473)	0.000	11.064 (6.324–19.357)	0.000
UIC (µg/L)								
<150 (Iodine deficiency)	1.319 (0.800–2.175)	0.277	1.405 (0.856–2.306)	0.179	1.001 (0.637–1.572)	0.997	0.979 (0.629–1.526)	0.927
150–500 (Iodine sufficiency)	1 (referent)		1 (referent)		1 (referent)		1 (referent)	
>500 (Iodine excess)	--	--	--	--	0.546 (0.071–4.167)	0.559	0.849 (0.113–6.361)	0.873

^1^ Multivariable models are adjusted for age, BMI, education, and smoking status. Abbreviations: UIC, urinary iodine concentration; SIC, serum iodine concentration.

**Table 6:** Binary logistic regression exploring the effect of serum iodine level on iodine status.

SIC (µg/L)	UIC < 150 µg/L				UIC > 500 µg/L			
	Unadjusted		Adjusted ^1^		Unadjusted		Adjusted ^1^	
	OR (95% CI)	*p*	OR (95% CI)	*p*	OR (95% CI)	*p*	OR (95% CI)	*p*
<72.4 (Quartile 1)	1.259 (1.044–1.519)	0.016	1.244 (1.031–1.502)	0.023	0.694 (0.294–1.637)	0.404	0.675 (0.286–1.596)	0.371
72.4–93.9 (Quartile 2 + Quartile 3)	1(referent)		1 (referent)		1 (referent)		1 (referent)	
>93.9 (Quartile 4)	1.001 (0.835–1.199)	0.992	1.000 (0.834–1.198)	0.997	1.869 (1.026–3.404)	0.041	1.866 (1.024–3.399)	0.042

^1^ Multivariable models are adjusted for age, BMI, education, and smoking status. UIC, urinary iodine concentration; SIC, serum iodine concentration.

## Data Availability

The data presented in this study are available on request from the corresponding author. The data are not publicly available due to it involves personal information.

## Supplementary Materials

The following supporting information can be downloaded at: https://www.mdpi.com/article/10.3390/nu15163555/s1, Table S1: Poisson regression exploring the effect of serum iodine level and urinary iodine level on thyroid diseases.

## References

[1] Zimmermann M.B. (2016). The Importance of Adequate Iodine during Pregnancy and Infancy. World Rev. Nutr. Diet..

[2] Alexander E.K., Pearce E.N., Brent G.A., Brown R.S., Chen H., Dosiou C., Grobman W.A., Laurberg P., Lazarus J.H., Mandel S.J. (2017). 2017 Guidelines of the American Thyroid Association for the Diagnosis and Management of Thyroid Disease During Pregnancy and the Postpartum. Thyroid.

[3] Zimmermann M.B. (2009). Iodine deficiency. Endocr. Rev..

[4] Zimmermann M.B., Gizak M., Abbott K., Andersson M., Lazarus J.H. (2015). Iodine deficiency in pregnant women in Europe. Lancet Diabetes Endocrinol..

[5] Hess S.Y., Ouedraogo C.T., Young R.R., Bamba I.F., Stinca S., Zimmermann M.B., Wessells K.R. (2017). Urinary iodine concentration identifies pregnant women as iodine deficient yet school-aged children as iodine sufficient in rural Niger. Public Health Nutr..

[6] Wang Z., Liu P., Su X., Zou S., Song J., Liu S. (2018). A Comparison of Iodine Status in Children and Pregnant Women After a Policy Change in the Iodized Salt Standard in Shanghai, China. Biol. Trace Elem. Res..

[7] Fan L., Su X., Shen H., Liu P., Meng F., Yan J., Lei Z., Zhang S., Gu Y., Liu S. (2017). Iodized salt consumption and iodine deficiency status in China: A cross-sectional study. Global Health J..

[8] Andersson M., de Benoist B., Delange F., Zupan J. (2007). Prevention and control of iodine deficiency in pregnant and lactating women and in children less than 2-years-old: Conclusions and recommendations of the Technical Consultation. Public Health Nutr..

[9] Zimmermann M.B., Andersson M. (2012). Assessment of iodine nutrition in populations: Past, present, and future. Nutr. Rev..

[10] Zimmermann M.B. (2004). Assessing iodine status and monitoring progress of iodized salt programs. J. Nutr..

[11] Ma Z.F., Skeaff S.A. (2014). Thyroglobulin as a biomarker of iodine deficiency: A review. Thyroid.

[12] Yu S., Yin Y., Cheng Q., Han J., Cheng X., Guo Y., Sun D., Xie S., Qiu L. (2018). Validation of a simple inductively coupled plasma mass spectrometry method for detecting urine and serum iodine and evaluation of iodine status of pregnant women in Beijing. Scand. J. Clin. Lab. Investig..

[13] Michalke B., Witte H. (2015). Characterization of a rapid and reliable method for iodide biomonitoring in serum and urine based on ion chromatography-ICP-mass spectrometry. J. Trace Elem. Med. Biol..

[14] Yu S., Wang D., Cheng X., Zhang Q., Wang M., Guo H., Yu B., Zhang X., Xia L., Sun D. (2020). Establishing reference intervals for urine and serum iodine levels: A nationwide multicenter study of a euthyroid Chinese population. Clin. Chim. Acta.

[15] Cui T., Wang W., Chen W., Pan Z., Gao S., Tan L., Pearce E.N., Zimmermann M.B., Shen J., Zhang W. (2019). Serum Iodine Is Correlated with Iodine Intake and Thyroid Function in School-Age Children from a Sufficient-to-Excessive Iodine Intake Area. J. Nutr..

[16] Jin X., Jiang P., Liu L., Jia Q., Liu P., Meng F., Zhang X., Guan Y., Pang Y., Lu Z. (2017). The application of serum iodine in assessing individual iodine status. Clin. Endocrinol..

[17] Pan Z., Cui T., Chen W., Gao S., Pearce E.N., Wang W., Chen Y., Guo W., Tan L., Shen J. (2019). Serum iodine concentration in pregnant women and its association with urinary iodine concentration and thyroid function. Clin. Endocrinol..

[18] Konig F., Andersson M., Hotz K., Aeberli I., Zimmermann M.B. (2011). Ten repeat collections for urinary iodine from spot samples or 24-h samples are needed to reliably estimate individual iodine status in women. J. Nutr..

[19] Li C., Peng S., Zhang X., Xie X., Wang D., Mao J., Teng X., Shan Z., Teng W. (2016). The Urine Iodine to Creatinine as an Optimal Index of Iodine During Pregnancy in an Iodine Adequate Area in China. J. Clin. Endocrinol. Metab..

[20] Chen W., Li X., Guo X., Shen J., Tan L., Lin L., Wu Y., Wang W., Wang W., Bian J. (2017). Urinary iodine excretion (UIE) estimated by iodine/creatinine ratio from spot urine in Chinese school-age children. Clin. Endocrinol..

[21] Iacone R., D’Elia L., Guida B., Barbato A., Scanzano C., Strazzullo P. (2018). Validation of daily urinary creatinine excretion measurement by muscle-creatinine equivalence. J. Clin. Lab. Anal..

[22] Hilderink J.M., van der Linden N., Kimenai D.M., Litjens E., Klinkenberg L., Aref B.M., Aziz F., Kooman J.P., Rennenberg R., Bekers O. (2018). Biological Variation of Creatinine, Cystatin C, and eGFR over 24 Hours. Clin. Chem..

[23] Blazewicz A., Klatka M., Dolliver W., Kocjan R. (2014). Determination of total iodine in serum and urine samples by ion chromatography with pulsed amperometric detection—Studies on analyte loss, optimization of sample preparation procedures, and validation of analytical method. J. Chromatogr. B.

[24] Haddow J.E., Palomaki G.E., Allan W.C., Williams J.R., Knight G.J., Gagnon J., O’Heir C.E., Mitchell M.L., Hermos R.J., Waisbren S.E. (1999). Maternal thyroid deficiency during pregnancy and subsequent neuropsychological development of the child. N. Engl. J. Med..

[25] Zimmermann M.B. (2009). Iodine deficiency in pregnancy and the effects of maternal iodine supplementation on the offspring: A review. Am. J. Clin. Nutr..

[26] Dosiou C., Medici M. (2017). Management of endocrine disease: Isolated maternal hypothyroxinemia during pregnancy: Knowns and unknowns. Eur. J. Endocrinol..

[27] Wu Y., Yang J., Su Q., Gu H., Qin L. (2023). Urinary iodine concentration and its associations with thyroid function in pregnant women of Shanghai. Front. Endocrinol..

[28] Andersen S.L., Sorensen L.K., Krejbjerg A., Moller M., Laurberg P. (2014). Challenges in the evaluation of urinary iodine status in pregnancy: The importance of iodine supplement intake and time of sampling. Eur. Thyroid J..

[29] Andersen S.L. (2015). Iodine status in pregnant and breastfeeding women: A Danish regional investigation. Dan. Med. J..

[30] Ainy E., Ordookhani A., Hedayati M., Azizi F. (2007). Assessment of intertrimester and seasonal variations of urinary iodine concentration during pregnancy in an iodine-replete area. Clin. Endocrinol..

[31] Kung A.W. (2007). Iodine nutrition of pregnant and lactating women in Hong Kong, where intake is of borderline sufficiency. Public Health Nutr..

[32] Rezvanian H., Aminorroaya A., Majlesi M., Amini A., Hekmatnia A., Kachoie A., Amini M., Emami J. (2002). Thyroid size and iodine intake in iodine-repleted pregnant women in Isfahan, Iran. Endocr. Pract..

[33] Arrizabalaga J.J., Larranaga N., Espada M., Amiano P., Bidaurrazaga J., Latorre K., Gorostiza E. (2012). Changes in iodine nutrition status in schoolchildren from the Basque Country. Endocrinol. Nutr..

