# Supplementary material for: Serum Iodine as a Potential Individual Iodine Status Biomarker: A Cohort Study of Mild Iodine Deficient Pregnant Women in China
Source: Nutrients. 2023 Aug 11;15(16):3555. doi: 10.3390/nu15163555 (PMC10460065; doi:10.3390/nu15163555)
Supplement: Supplementary file 1 [file nutrients-15-03555-s001.zip › nutrients-2540701-supplementary.pdf]

Supplementary Information

Serum Iodine as a Potential Individual Iodine Status Biomarker: A Cohort Study of Mild Iodine Deficient Pregnant Women in China

Xueqing Li, Pengcheng Tu, Simeng Gu, Zhe Mo, Lizhi Wu, Mingluan Xing, Zhijian Chen and Xiaofeng Wang *

Department of Environmental Health, Zhejiang Provincial Center for Disease Control and Prevention, 3399 Binsheng Road, Hangzhou 310051, China; xqli@cdc.zj.cn (X.L.); pchtu@cdc.zj.cn (P.T.); smgu@cdc.zj.cn (S.G.); zhmo@cdc.zj.cn (Z.M.); lzhwu@cdc.zj.cn (L.W.); mlxing@cdc.zj.cn (M.X.); zhjchen@cdc.zj.cn (Z.C.)

* Correspondence: xfwang@cdc.zj.cn; Tel.: +86-0571-87115225

Table S1. Poisson regression exploring the effect of serum iodine level and urinary iodine level on thyroid diseases.

	**Hypothyroxinemia**				**Thyrotoxicosis**			
	**Unadjusted**		**Adjusted[1]**		**Unadjusted**		**Adjusted[1]**	
	**OR (95% CI)**	***P***	**OR (95% CI)**	***P***	**OR (95% CI)**	***P***	**OR (95% CI)**	***P***
SIC (μg/L)								
<72.4 (Quartile 1)	**9.046 (5.288-15.473)**	**0.000**	**8.165 (4.787-13.927)**	**0.000**	0.403 (0.117-1.390)	0.150	0.412 (0.119-1.423)	0.161
72.4-93.9 (Quartile 2 +Quartile 3)	1(referent)		1(referent)		1(referent)		1(referent)	
>93.9 (Quartile 4)	0.380 (0.111-1.303)	0.380	0.392 (0.114-1.346)	0.392	**10.405 (6.014-18.005)**	**0.000**	**10.425 (6.024-18.042)**	**0.000**
UIC (μg/L)								
<150 (Iodine deficiency)	1.007 (0.998-1.017)	0.133	1.006 (0.997-1.016)	0.184	1.154 (0.163-8.165)	0.886	1.146 (0.160-8.226)	0.892
150-500 (Iodine sufficiency)	1(referent)		1(referent)		1(referent)		1(referent)	
>500 (Iodine excess)	1.016 (1.008-1.024)	0.418	0.982 (0.944-1.021)	0.359	1.191 (0.165-8.582)	0.862	1.170 (0.161-8.522)	0.877

[1] Multivariable models are adjusted for age, BMI, education and smoking status.

Abbreviations: UIC, urinary iodine concentration; SIC, serum iodine concentration.
